# Return to Galileo? The Inquisition of the International Narcotic Control Board

**DOI:** 10.1186/1477-7517-5-16

**Published:** 2008-05-07

**Authors:** Dan Small, Ernest Drucker

**Affiliations:** 1 Department of Medicine, University of British Columbia, Vancouver, Canada; 2 Director, PHS Community Services Society, Vancouver, Canada; 3 Montefiore Medical Center, Albert Einstein College of Medicine, NYC, USA; 4 Columbia University, Mailman School of Public Health, NYC, USA

## Abstract

Nearly 400 years after Galileo Galilei of Florence was arraigned and convicted of suspected heresy by the ten member Congregation of the Holy Office (Inquisition), the International Narcotic Control Board (INCB) is similarly inserting itself into matters pertaining to innovations in healthcare and the public health response to addiction throughout the world. Like that earlier Inquisition of 1633 that convicted Galileo of heresy for holding that the sun is the centre of the universe with the earth revolving around it (in contradiction to church doctrine of the time) the INCB and its thirteen-member panel, now rails against any evidence out of sync with the established doctrine of the war on drugs – particularly those innovations in public health called harm reduction.

The latest healthcare and harm reduction practices to attract the ire of the INCB Inquisition are elements of Canada's most effective and innovative measures to minimize the harms of drugs in Vancouver – supervised injection facilities and, recently, the potential establishment of supervised inhalation rooms – along with the long established practice of providing safer mouthpieces for pulmonary inhalation in British Columbia. This is particularly significant as it comes in the midst of a crucial battle between municipal and provincial authorities in BC with the federal government in Ottawa, which seems determined to undermine all the most effective HR programs that are the result of years of steady local and governmental support in Vancouver and now threatens to derail all these programs and spread doubt about their usefulness despite the overwhelmingly positive findings of serous research.

## 

The Grand Inquisitor of the INCB Board, Chairman Dr. Philip Emafo, makes it his practice to issue stern warnings to Canada and all progressive countries that make HR their national policy and innovate HR practices. On behalf of the INCB Inquisition, Emafo pronounces that all countries must abandon the defense and practice of the dark arts of harm reduction and (like Galileo) must also publicly recant, condemning their leading population health initiatives in addiction work. If they do not, then the countries in question are accused of drug policy heresy.

Ironically, while the INCB was proclaiming its newest round of objections to evidenced based approaches to addiction in the first week of March 2008, at the very same time the Pontifical Academy of Sciences, with its headquarters in the Holy See under the direct protection of the Supreme Pontiff was helping to disavow the Inquisitions of Galileo four centuries ago, momentously announcing its plans to erect a statue of Galileo in Vatican City. Will the INCB likewise see the error of its ways and recant its own betrayal of the health and human rights of people with serious addictions? Today we are at a cross roads in Canada – will we defend the truth of evidence-based approaches to the pandemic of addiction, by the light of the lessons learned from this history, or return to the darkness of a time we thought long past?

*"Whereas you, Galileo, son of the late Vincenzio Galilei, Florentine, aged seventy years, were denounced to this Holy Office in 1615 for holding as true the false doctrine taught by some that the sun is the center of the world and motionless and the earth moves even with diurnal motion; for having disciples to whom you taught the same doctrine; for having been in correspondence with some German mathematicians about it; for having published some letters entitled On Sunspots, in which you explained the same doctrine as true...We condemn you to formal imprisonment in this Holy Office at our pleasure. As a salutary penance we impose on you to recite the seven penitentiary Psalms once a week for the next three years. And we reserve the authority to moderate, change, or condone wholly or in part the above-mentioned penalties and penances. This we say, pronounce, sentence, declare, order, and reserve by this or any other better manner or form that we reasonably can or shall think of. So we the undersigned Cardinals [Inquisitors] pronounce" *(Proceedings of the Inquisition of 1633 pp. 288–291)[1]

And so it was, in the presence of instruments of torture and under the formal threat of torture, Galileo was interrogated by the Inquisition (a judicial body of ten Cardinals) beginning on 12 April 1633 and concluding on 21 June 1633 following which he was forced to recant his heretical views about the nature of the universe on 22 June 1633 [1]. The nature of the dispute pertained to the traditional notion of the earth as geostatic (motionless) and geocentric (at the centre of the cosmos). The view that the earth laid motionless at the centre of the universe represented the popular wisdom of the day and had been espoused since the time of Greek philosopher Aristotle (384 BC – 322 BC) and later formalized by Greek astronomer Ptolemy (81 to 161 A.D.).

With regard to the accepted healthcare responses to addiction, we, too, appear to be in the midst of a metaphoric holy war between evidence and belief. But instead of dispute about doctrine regarding the movements of heavenly bodies, we are embroiled in a momentous struggle about the established doctrine of the war on drugs – a universally failed war militantly espoused by the United States and its allies – most cravenly by the Holy Office of Inquisition, the International Narcotic Control Board (INCB) – the UN body charged with the responsibility for maintaining accordance with drug control treaties and, in so doing, ensuring "adequate supplies of drugs are available for medical and scientific uses and that the diversion of drugs from licit sources to illicit channels does not occur" [2]. In fact, the treaties underlie the work of the INCB may better examined as religious texts and, as such, the UN may be an inherently challenging area in which to discuss evidence [3,4]. But often the Board uses its global pulpit to stifle innovation and intimidate legitimate public health innovators in all countries that do not conform to the bankrupt drug wars doctrine stemming back "as far as the League of Nations" [2].

On Wednesday 5 March 2008, the head of the International Control Board Dr. Philip Emafo issued another edict on drug addiction from his comfortable position on the summer side of life at his office in Vienna[5]. Perched on high in his wingback chair, the INCB head, upon review of the day's newspaper clippings, once again took it upon himself to criticize the work of healthcare practitioners a world away, this time turning his attention to the Vancouver Island Health Authority. Ironically, plans to erect statue of Galileo in Vatican City were announced the very same week on 7 March 2008 [6]. The statue was commissioned by the Pontifical Academy of Sciences. The Pontifical Academy is a body comprised of eighty internationally acclaimed academics elected from existing members and formally nominated by the Pope. The history of the institution can be traced to 1603 when it was the Academy of Lincei, one of the first academies of its kind, of which Galileo was a member. This scientific institution was renewed in 1847 by Pope IX under a new name as the Pontifical Academy of the New Lincei and later renamed under Pope Pius X1 in 1936 under its current configuration as the Pontifical Academy of Sciences. Today, it enjoys the protection by the reigning Pope and maintains its headquarters in the Vatican where its members assemble every year in the Casina of Pius IV [7].

Of course, science and beliefs need not be incompatible as Pope Paul II stated in a speech in 1992 where he officially recognized the mistakes of the church for having convicted Galileo for believing that the earth was not the centre of the universe and that it revolved around the sun [6]. Still earlier in 1981, Pope Paul II, a pontiff from the homeland of Copernicus, had put in place a commission to study the learning opportunities for theologians from the treatment of Galileo.

The current pontiff, Pope Benedict XVI, has publicly praised the contributions of Galileo.

However, these valuable lessons about the relationship between belief and the advancement of human knowledge appear to have been lost on the INCB, which rigidly clings to the outmoded scriptures of the war on drugs and maintains an open hostility towards evidenced based approaches to addressing addiction. In the universe of the INCB, the sun still revolves around the earth at the centre of the cosmos and any opinions to the contrary are deemed as Heretical. While we, and others, have pointed out that the INCB appears "closed to reason", it does not appear to be either evidence or any thoughtful logic that guides the actions of this body and its head [8,9]. Like the Inquisition some four centuries earlier, it seems more accurate to consider the actions of the INCB, and its Inquisitor General, Dr. Emafo, although he is ironically trained as scientist, as leading a metaphoric holy war in the realm of addiction. In this holy war, the Board's doctrine is American-centric: with its policies revolving around the United States federal drug policy with enforcement, treatment and prevention as central scriptures:

"The Board welcomes the United States Government's unequivocal policy position against any form of legalization of the non-medical use of drugs" [10] (p. 10).

There is no book of harm reduction allowed in the United States bible of drug policy and it fiercely opposes such innovations that turn, instead, around local realities and the need for evidenced based population health responses. Like the Church's ferocious attack on Copernican heliocentrism championed by Galileo Galilei in 1633, Dr. Emafo and his inquisitors highlight scriptural heresy for the attention of the Holy Church of the INCB, the United States – path breaking programs such as heroin maintenance, supervised injection facilities[11] or mouthpieces[5] for people addicted to crack cocaine.

The most recent innovation relate to the rapid growth in world markets for stimulants and other drugs that are used by inhalation – in part a reflection of growing awareness of the hazards of injecting and its risks for transmission of deadly infectious diseases – HIV and HCV. The fact remains that no matter how much we may wish it not to be, there is a pandemic of smoking and snorting illicit drugs, such as crack cocaine and crystal methamphetamine, and that this activity rivals injection drug use in many countries. But the sharing of implements for smoking or inhaling illicit drugs is also now known to be a risk factor for HCV or HIV [12,13]. Furthermore, it may be the case that sharing of inhalation equipment may link intravenous drug using and non-injecting drug using populations.

One of primary risks associated with crack smoking is posed by the use of inadequate pipes. Most crack users cannot afford commercially purchased pipes so they make use of metal tubing such as car antennas that transmit heat when a flame is applied to the end to vaporize the drug [14]. This technique can result in burned or blistered lips. Crack pipes are frequently shared; the pipes are passed from one person to the other with each individual smoking some of the drug [14]. When this paraphernalia is shared, bodily fluids such as saliva or blood carrying HCV can travel between persons [12]. The Hepatitis C (HCV) virus is a significant cause of liver damage in the world and the related disease processes including fibrosis, cirrhosis and hepatocellular carcinoma [15]. Of those individuals affected with HCV, between sixty and eighty percent develop chronic hepatitis leading to significant morbidity and mortality. The smoking of crack cocaine causes blisters, sores and cuts in the mouth which may also lead to the transmission of HIV [13]. Once blisters or cuts are created by inadequate pipes or filters, then a further risk may also be posed through the transmission of infected blood through oral sexual activity and the sharing of pipes [14].

A second risk is created by the utilization of fragile glass pipes as these present risks in that they crack when heated or dropped [14]. A jagged glass pipe can cut the lips of a drug user thereby presenting a risk for infection through exposure to blood when crack pipes are shared between persons. As a harm reduction measure, a safer pipe, made of heat resistant material such as Pyrex, can be substituted to reduce likelihood of cuts from an inferior glass pipe that is prone to cracking under heat.

A third risk is posed by the use of inadequate filters used by crack smokers [14]. Furthermore, drug users use copper or steel wool, such as brillo pads, as filters for the pipes. These compact pieces of steel wool are designed for cleaning pots and pans and often contain detergents. At times, particles of steel wool break off from these makeshift filters and, at times, are inhaled and cut or burn the drug users' lips. Smoking stimulants such as crystal meth-amphetamine or crack cocaine may also effect the pulmonary system leading to lung damage, infection, pulmonary edema or respiratory failure [16-23]. The filter presents an obvious place to intervene with a harm minimization strategy by providing a safer replacement.

While the risk of death due to injection of heroin is well established, fatal overdoses are not limited to injection [24]. Snorting (intranasal ingestion) or smoking (pulmonary inhalation) of heroin can be lethal [25]. Methods of inhaling drugs can also introduce hazardous concentrations of opiate in the blood stream. Risk of death from inhalation (snorting or smoking) may be further increased when other drugs, such as alcohol, are simultaneously ingested [24,25]. Lethality may be further amplified by compromised physical health such as decreased organ function.

There are practical population health responses to the risks outlined above that can mitigate the dangers of snorting and smoking illicit drugs. First, flexible and durable mouthpieces need to be provided to cover the tip of the pipes so that drug users' lips are not blistered or cut. Secondly, particularly dangerous pipes, such as those made out of metal or glass, need to be replaced so that the harms posed by cuts or burns are reduced and in turn reduce risk of the transmission of hepatitis or HIV. Thirdly, the primitive steel wool filters need to be replaced with a durable and safe substitute that can be inserted into the end of the pipe without danger of breaking down and posing risks of inhaling chemical detergents and metal or being cut by shards. Fourth, the potential overdoses from intranasal or ingestion of stimulants (e.g. heroin, crack cocaine, crystal methamphetamine) could be mitigated through the provision of a supervised inhalation facility. A supervised inhalation facility would provide the opportunity for a highly marginalized group of drug users to be brought into the doorway of healthcare where they can have access to harm reduction, preventive population based health innovations, treatment, detox and supported housing.

In Canada, in partnership with the Vancouver Coastal Health Authority, the PHS Community Services Society operates a Supervised Injection Facility (SIF). While the SIF and other programs provide a desperately needed entry level of health engagement for people with active addictions who inject illegal drugs, there are still several thousand people in British Columbia that are addicted to illegal drugs (such as crack cocaine or crystal methamphetamine) that are smoked. Currently, this group of people is still forced to use drugs in open public spaces and unsafe environments where access to housing, health and treatment services are minimal.

The aim of the harm minimization efforts such as the provision of mouthpieces or a supervised inhalation pilot would be to match the positive effects of the supervised injection initiative by reaching a target group that is otherwise unengaged in any form of medical or support services in order to reduce the harms associated with smoking crack cocaine and crystal methamphetamine while dramatically reducing public disorder and open drug use. In British Columbia, medical and public health authorities and practitioners had established a standard of care for one group with serious needs (those who inject drugs), but inadvertently excluded the needs of an equally needy target group (those who inhale or smoke drugs). In many jurisdictions including Canada, the distribution of people with serious addictions who inject is roughly the same as those who inhale. In some settings, the numbers of those who inhale drugs are overtaking those who inject.

The Mayor of Victoria Alan Lowe [26], the Victoria Island Health Authority[26], the Chief Medical Health Officer of B.C. (Dr. Perry Kendall)[27,28] and the Vancouver Coastal Health Authority[29] share the view that there needs to be a variety of strategies to engage the equally marginal group of people living with active additions who smoke drugs such as crack cocaine or crystal methamphetamine. In fact, provisions for the purchase of mouthpieces have been made in provincial budgets since 2007. Individual health authorities determine the provision of these harm reduction items.

There is also a need to go still further in reaching people with addiction to smoking stimulants such as crack cocaine and crystal methamphetamine. There is a need to establish a supervised inhalation pilot in British Columbia. The international standard of practice for safer consumption rooms is to operate supervised **injection **initiatives together with supervised **inhalation **programs. By way of example, there are 12 safe consumption facilities in Switzerland. Of these, eight have spaces for injection and inhalation. Similarly, there are 22 safe consumption facilities in the Netherlands. All of them have space for both injection and inhalation. In Germany, there are 25 consumption rooms with 13 providing space for inhalation as well as injection[30].

In the Canadian setting, we have established a standard of care for one group with serious needs (those who inject drugs), but inadvertently excluded the needs of an equally needy target group (those who inhale or smoke drugs). We believe that a supervised inhalation room needs to be opened as soon as possible to resolve this disparity by reaching the equally marginal group of people living with active additions who smoke drugs such as crack cocaine or crystal methamphetamine. A second research pilot needs to be launched that examines the ability of a supervised inhalation initiative aimed at reaching a target group that is otherwise unengaged in any form of medical or support services in order to reduce the harms associated with smoking crack cocaine and crystal methamphetamine while dramatically reducing public disorder and open drug use.

## Concluding thoughts: a return to Galileo

Far removed from the suffering of people with addictions in the shadows of life, the INCB Grand Inquisitor judges adherence to drug policy scriptures and keeps a watchful eye out for heresy. In formal terms, the INCB has all the ferocity of a papier-mâché tiger in matters of public health. Apparently ferocious, upon careful inspection, this political body poses no serious threat to legitimate initiatives aimed at welcoming people with serious addictions into the doorway of healthcare, like the provision of mouthpieces to combat HCV or supervised injection facilities to combat epidemics of HIV and overdose deaths. But the real danger they pose is through insidious political influence – giving comfort to the local enemies of such programs and, more significantly, offering a seemingly (if not actually) authoritative international voice for retrograde policies that fly in the face of both scientific evidence and humane concerns.

Through their totally illegitimate political influence, the Inquisitors of the INCB now threaten to undermine the comprehensive approach to addiction in Canada – an approach that has become an international beacon of progress. This approach is based on the principles and best practices of harm reduction and includes some of the best and most innovative treatment and prevention programs for addiction in the world – e.g. easy to access to detoxification, supervised injection facilities, needle distribution programs, pharmaceutically assisted therapies (methadone, heroin, stimulant replacement under a physician's care), safer crack-pipe mouthpieces, effective prevention and thoughtful enforcement then too many parents will be saying their final goodbye to their son or daughter at the funeral home due to overdose or the unfortunate reach of the Hep C and AIDS pandemic. Through these efforts Canadian healthcare professionals, now have access to the best tools for the medical tool-belt, even if it contradicts the scripture of the American war on drugs.

The real question here is how we will return to the lessons of Galileo. Debate pertaining belief, the evidence base and the best way to move forward with best practices for addiction medicine and healthcare are, of course, legitimate. But we have to remember that no single tree grows to heaven when it comes to addiction. There is not only one approach and unsure cures are sometimes better than no cures at all. We need many approaches and innovations to approach the various forms of serious and persistent drug addiction. Fortunately, the INCB, we have to remember, is not the United Nations. Nor do they represent the United Nations. The creation of the INCB can be traced to three treaties, the Single Convention on Narcotic Drugs (1961), the Convention on Psychotropic Substances (1971) and the United Nations Convention against Illicit Traffic in Narcotic Drugs and Psychotropic Substances (1988)[2] The INCB has a limited responsibility to ensure that adequate supplies of narcotic drugs are available in the world for medical and scientific use and identifying limitations in controls that lead to the sale, use or manufacturing of illicit drugs. The INCB is primarily concerned with the international and national monitoring and management of illicit and licit drugs. Despite media reports, the INCB is **not **the United Nations. The United Nations readily recognizes the need for efficacious and evidenced-based action with respect to the AIDS pandemic and, as such, the United Nations General Assembly **unanimously **publicly declared the importance of harm reduction on 2 June 2006 [31].

Far away from comfort of the comfortable offices of the INCB Grand Inquisition in Vienna; healthcare, housing and service providers are earnestly attempting innovations amidst the shards of broken dreams. We must not falter, despite this attention from the shadowy pressure from the INCB, to work towards developing evidenced based healthcare innovations in response to each new phase of addiction as they unfold.

In contrast to Galileo who aimed his telescope at the skies, those of us who walk down the old and dusty road of healthcare are focusing on more earthly problems and, in so doing, trying to help alleviate the burden of suffering for real people, their families, and society at large. In the geostatic world of the INCB Inquisitors, with the United States drug policy at the centre of the cosmos, the world may indeed be either black or white. But for those healthcare practitioners and service providers trying to cobble together effective strategies to address the pandemic of addiction, there are, by necessity, many colours in the spectrum of social problems.

In the moral borderland of addiction, it is sometimes easier to burn metaphoric bridges than to build them. And in the world of the INCB, perhaps, those countries and healthcare practitioners who practice harm reduction are expected to prepare a solemn recantation such as Galileo's:

"I, Galileo, son of the late Vincenzio Galilei of Florence, seventy years of age, arraigned personally for judgment, kneeling before you Most Eminent and Most Reverend Cardinal's Inquisitors-General against heretical depravity in all of Christendom, having before my eyes and touching my hands the Holy Gospels, swear that I have always believed, I believe now, and with God's help I will believe in the future all that the Holy Catholic and Apostolic Church holds, preaches, and teaches...I have been judged vehemently suspected of heresy, namely of having held and believed that the sun is the centre of the world and motionless and the earth is not the centre and moves...I, Galileo Galilei, have adjured as above, by my own hand" (Proceedings of the Inquisition of 1633 pp. 292–293) [1]

For the INCB Inquisitors, it is relatively easy, from afar, to condemn the earnest efforts of healthcare providers who attempt however possible to engage marginalized populations of people with addictions in the doorway of healthcare. The INCB appears hell-bent on trying to ignite political fires and this is sometimes disheartening for everyday people working at the local level. Perhaps, we are all naively traveling up a long and lonely stream promoting the idea that addiction is a matter for the Chief of Medicine rather than the Chief of Police and it is time for us to prepare our renunciation of all harm reduction for the INCB Inquisitors. But we think not.

After all – still under threat from the Inquisition – even Galileo got out the words of the need for truthfulness in science. And while there is no definitive proof that at this time he whispered, "Eppur si muove" ("And yet it **moves**")[32] he did write in a Letter to the Grand Duchess Christina in 1615:

"However, I do not think one has to believe that the same God who has given us **senses, language, and intellect **would want to set aside the use of these and give us by other means the information we can acquire them, so that we would deny our senses and reason even in the case of those physical conclusions which are placed before our eyes and intellect by our sensory experiences or by necessary demonstrations [emphasis added]"[1] (p.95).

In the face of the physical conclusions of harm reduction that have been placed before our eyes and intellect, will we in public health and medicine do any less and forgo (or recant) the evidence base seen by our own **"senses, language, and intellect"**? We think not-ever again!

## Competing interests

The authors declare that they have no competing interests.

## Authors' contributions

DS wrote the first draft. Both authors participated in the writing of the manuscript and approved the final version.
